# Surface solvation of Martian salt analogues at low relative humidities†

**DOI:** 10.1039/d1ea00092f

**Published:** 2022-01-25

**Authors:** Xiangrui Kong, Suyun Zhu, Andrey Shavorskiy, Jun Li, Wanyu Liu, Pablo Corral Arroyo, Ruth Signorell, Sen Wang, Jan B. C. Pettersson

**Affiliations:** a Department of Chemistry and Molecular Biology, Atmospheric Science, University of Gothenburg SE-41296 Gothenburg Sweden kongx@chem.gu.se janp@chem.gu.se; b MAX IV Laboratory, Lund University SE221-00 Lund Sweden; c Shaanxi Key Laboratory of Earth Surface System and Environmental Carrying Capacity, Northwest University Xi'an 710127 China; d Department of Chemistry and Applied Biosciences, ETH Zurich Zurich Switzerland

## Abstract

Salt aerosols play important roles in many processes related to atmospheric chemistry and the climate systems on both Earth and Mars. Complicated and still poorly understood processes occur on the salt surfaces when interacting with water vapor. In this study, ambient pressure X-ray photoelectron spectroscopy (APXPS) is used to characterize the surface chemical environment of Martian salt analogues originating from saline lakes and playas, as well as their responses to varying relative humidities. Generally, APXPS shows similar ionic compositions to those observed by ion chromatography (IC). However, XPS is a surface-sensitive method while IC is bulk-sensitive and differences are observed for species that preferentially partition to the surface or the bulk. Element-selective surface enhancement of Cl^−^ is observed, likely caused by the presence of SO_4_^2−^. In addition, Mg^2+^ is concentrated on the surface while Na^+^ is relatively depleted in the surface layer. Hence, the cations (Na^+^ and Mg^2+^) and the anions (Cl^−^ and SO_4_^2−^) show competitive correlations. At elevated relative humidity (RH), no major spectral changes were observed in the XPS results, except for the growth of an oxygen component originating from condensed H_2_O. Near-edge X-ray absorption fine structure (NEXAFS) measurements show that the magnesium and sodium spectra are sensitive to the presence of water, and the results imply that the surface is fully solvated already at RH = 5%. The surface solvation is also fully reversible as the RH is reduced. No major differences are observed between sample types and sample locations, indicating that the salts originated from saline lakes commonly have solvated surfaces under the environmental conditions on Earth.

## Abstract 2

Salt aerosols play important roles in many processes related to atmospheric chemistry and the climate systems on both Earth and Mars.

## Graphical abstract

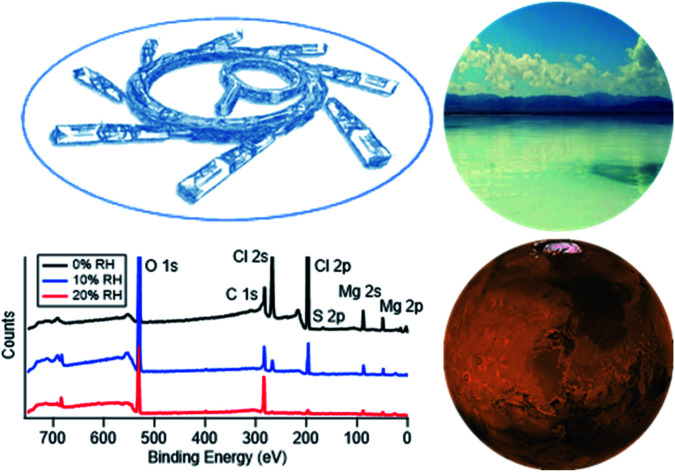

**Environmental significance**

Salt aerosols play important roles in many processes related to atmospheric chemistry and the climate system, and an important and dynamic source of salt aerosol is saline lakes and playas. The saline lakes and playas are also considered as a good terrestrial analogue for Mars. Sulfate and chloride component sampled playas exist in a highly hydrated form, like Martian salts. The identified hydrated Martian salts raise the question about the presence of liquid water on Mars. Here, we use ambient pressure X-ray photoelectron spectroscopy to study the surface chemical environment of saline lake and playa salts. We found that the high RH sensitivity is a common feature for these salts, indicating that under the typical environmental conditions on Earth and Mars the salt mixtures commonly have solvated surfaces.

## 1 Introduction

Salt aerosols play important roles in many processes related to atmospheric chemistry and the climate system,^1^ especially as active components in aerosol and cloud formations^2,3^ due to their high hygroscopicity.^4–9^ An important and dynamic source of salt aerosols is saline lakes and playas,^10,11^ which produce salt particles mixed with mineral dust that are transported over long distances.^5,7,12–18^ Furthermore, saline lakes and playas are becoming more relevant as they are continuously developing, particularly in the context of climate change.^19^

Salt particles are also associated with haze formation in more populated regions. For example, the formation of secondary inorganic aerosols (SIAs) in Shanghai is promoted by salt aerosols after long-distance transportation from East Asian desert regions.^17^ The formed haze is more harmful to human health compared to the primarily emitted dust and salt aerosols.^20^ Also, during transportation the salt–dust mixture can undergo heterogeneous reactions and transform into materials with higher hygroscopicity.^21,22^ It has been reported that the Cl-rich dust from playas can react with N_2_O_5_ and produce atmospheric reactive chlorine that facilitates tropospheric ozone formation.^23^ Thus, facilitated by long-range transport and heterogeneous pathways, the playa-originated salt particles impact the air quality and human health.^24^ As one of the largest regions of saline lakes and playas on Earth,^25^ the Qaidam (meaning *salt flat* in Mongolian) Basin is the sampling region of this study. The Qaidam Basin is located in the Qinghai–Tibet plateau, and the specific geographic conditions make the location extremely sensitive to climate change.^25^

The saline lakes and playas, especially in the Qaidam Basin, are also considered as a good terrestrial analogue for Mars, as recent explorations and studies revealed that the Martian crusts have similar compositions.^26–29^ About one-third of the Martian surface is believed to have been covered by oceans and lakes in the early stage of Mars development.^30^ The huge water bodies dissolved a large quantity of minerals that were precipitated and formed evaporates and salt deposits during Martian drought periods.^31^ Sulfates, chlorides and carbonates are the common salt compositions deposited both in the Qaidam Basin and on Mars.^32–35^ Wang and coworkers found that the sulfate and chloride components sampled from the Qaidam Basin exist in a highly hydrated form in spite of the hyper-arid conditions, similar to the Martian environment.^36–38^ Highly hydrated salts have been identified on Mars, which raises the question about the presence of liquid water on Mars.^37,39,40^ Also, the hydrated forms of sulfate might be the cause of present-day topographic changes observed on Mars.^41^ Another recently discovered phenomenon on Mars is the recurring slope lineae (RSL),^42^ which is hypothesized to mainly be caused by hydrous chlorides and oxychlorine salts through deliquescence.^43,44^ However, the water vapor levels required for deliquescence are much higher than those in the present Martian atmosphere, thus the RSL formation mechanism and the roles of salts need to be further understood. The knowledge gap of salt–water interactions has limited our understanding about the Martian environment, and a more comprehensive picture on the governing mechanisms may greatly enhance our understanding and accelerate Martian exploration.

Given these aspects, the Qaidam salts can be regarded as proxies for inland-originated atmospheric salt particles on Earth and salts on Mars. A better understanding of the interactions between gas phase water and the Qaidam salts will shed light on the roles of salt mixtures in the environment on both Earth and Mars. Another merit of studying saline lake salts is that these salts are relatively simple in terms of low biogenic activities and low levels of organics, which serves as an excellent bridge between laboratory pure systems and realistic complex systems. In this study, we use a state-of-the-art experimental approach, ambient pressure X-ray photoelectron spectroscopy (APXPS), to study the influence of relative humidity (RH) on the surface chemical environment of Qaidam salts. The near-edge X-ray absorption fine structure (NEXAFS) approach is also used to probe the local environment on the salt surface under interactions with water vapor. This work is intended as an explorative APXPS study on natural salt mixtures with the idea of verifying the feasibility of studying natural substances with APXPS. The salt samples were purposely kept in their original states with minimum processing. Further studies, *e.g.*, on genuine Martian samples in a decade or so, could follow a similar strategy to identify the differences and similarities between Earth and Mars.

## 2 Materials and methods

### 2.1 Sampling sites and sampling methods

**Fig. 1:**
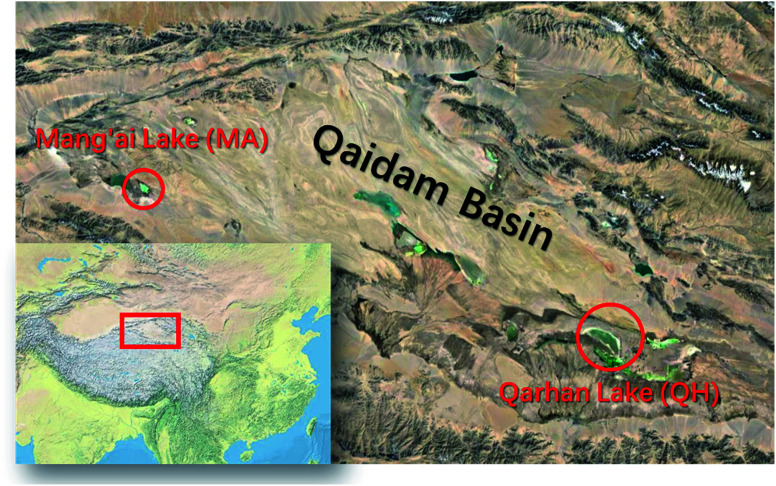
Sampling locations, Mang-ai Lake (MA) and Qarhan Lake (QH), in the Qaidam Basin. The sampling was carried out during 11^th^–14^th^ September 2020.

The salt samples were collected from two saline lakes in the Qaidam Basin (Fig. 1). The Qaidam paleolake was migrating from the west (the Mang'ai Lake) to the east (the Qarhan Lake),^25^ and salt mixtures from these two lakes are selected to represent lakes in different development stages. The Qarhan (QH) Lake is located in the south-central part of the Qaidam Basin. It is one of the largest inland saline lakes in the world and the largest soluble potassium magnesium salt deposit in China.^45^ The Mang'ai (MA) Lake is located on the western edge of the Qaidam Basin, lying on the Kunlun Mountain in the south and Altun Mountain in the north. It is a salt pond continuously recharged by underground brine.

The acquired brines were taken from 2–5 cm below the lake surface, and then filtered by using filter paper (Beimu, GB/T1914-2017, pore size < 20 μm) within 48 hours after sampling. The lakebed salts were collected at the same sampling points as brines. The salt crusts were collected from nearby playa fields. All the samples were stored in polyethylene bottles and sealed with Parafilm membrane, and then stored in a fridge at 4 °C.

### 2.2 Ion chromatography

Saturated solutions of lakebed and crust salts were prepared by dissolving them in ultrapure water. These saturated solutions, including sampled brines, were diluted by a factor of 1000 to meet the upper detection limits of ion chromatography (IC). The diluted solutions were then filtered through 0.22 μm water filter membranes. The cations (Na^+^, K^+^, Mg^2+^ and Ca^2+^) and anions (Cl^−^ and SO_4_^2−^) were measured by using the Dionex AQUION IC system (Thermo Scientific, USA). The cations were analyzed using CS12A IC columns (Dionex IonPac, Thermo Fisher) with 30 mmol L^−1^ methanesulfonic acid eluent. The anions were analyzed by using AS11-HC IC columns (Dionex IonPac, Thermo Fisher) with 20 mmol L^−1^ KOH eluent.

### 2.3 Ambient pressure X-ray photoelectron spectroscopy

Ambient pressure X-ray photoelectron spectroscopy (APXPS) experiments were performed at the HIPPIE beamline of MAX IV in Sweden.^46^ Before and between measurements, the experimental cell was kept under high vacuum (1 × 10^−10^ mbar base pressure). The salt samples were dissolved in ultrapure water and deposited onto the sample holder by drop-casting. The solutions of the crust samples had a brownish color, due to some suspension of soil and dust particles. The lakebed and crust salt solutions were not filtered. After the samples had dried, they were transferred into the vacuum chamber. Water vapor was introduced from a glass water reservoir *via* a leak valve. The water source (Fluka TraceSelect Ultra; Water ACS reagent) was degassed using 3 freeze–pump–thaw cycles. During the experiments, the RH was varied by regulating the leak valve to change the partial pressure of water vapor. The sample temperature was kept at 16 °C. A summary of experimental RH, pressure and temperature values can be found in Table 1.

**Table 1:** Experimental temperatures, water vapor pressures, and RH used in the experiments

*T* (°C)	RH	Vapor pressure (mbar)
16 ± 0.5	0%	0
16 ± 0.5	4.4 ± 0.1%	0.8 ± 0.03
16 ± 0.5	9.4 ± 0.2%	1.7 ± 0.04
16 ± 0.5	20 ± 0.4%	3.6 ± 0.05
16 ± 0.5	30 ± 0.7%	5.4 ± 0.05

## 3 Results

### 3.1 Sample characterization

**Fig. 2:**
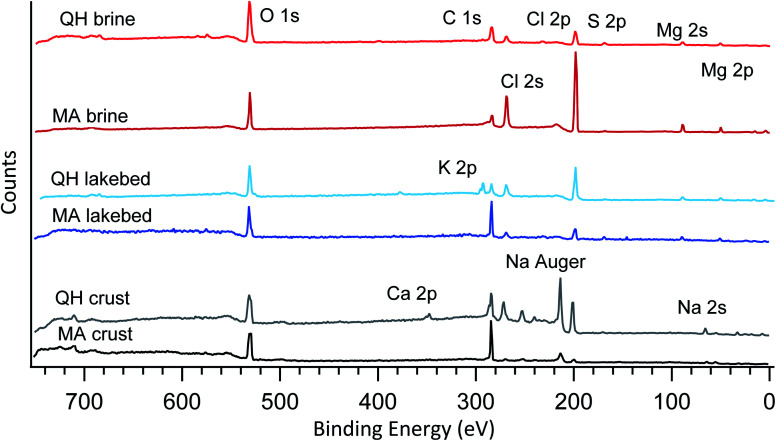
Broadband photoemission spectra of the six samples measured under vacuum. The photon energy used is 1200 eV. The major peaks are assigned and marked on the plot.

The samples were initially characterized by XPS in vacuum with broadband photon energies. The resulting photoemission spectra are shown in Fig. 2. No major charging or differential charging effects were observed as the thin samples had good electrical conductivity. During the measurements, no decay or formation of any species arose from the radiation exposure, indicating that the sample is not sensitive to the X-ray beam. The main elements observed include oxygen (O 1s), carbon (C 1s), chlorine (Cl 2s and Cl 2p), sulfur (S 2p), magnesium (Mg 2s and Mg 2p), sodium (Na 2s) and calcium (Ca 2p). These identified elements agree well with the ion chromatography results shown in Table 2.

**Table 2:** Ionic molar composition (mol L^−1^) measured by IC

	Na^+^	K^+^	Mg^2+^	Ca^2+^	Cl^−^	SO_4_^2−^
QH brine	0.221	0.111	3.701	0.009	8.795	0.160
MA brine	1.872	0.180	2.256	0.008	6.090	0.653
QH lakebed	0.412	0.865	2.067	0.004	6.244	0.040
MA lakebed	6.075	0.058	0.394	0.001	7.479	0.124
QH crust	5.391	0.028	0.008	0.032	6.090	0.050
MA crust	5.467	0.024	0.012	0.001	6.156	0.016

The six samples are grouped into three types (brines, lakebed salts and crust salts) as shown in Fig. 2. In the crystalized brine samples, O 1s, C 1s, Cl 2p, Cl 2s, S 2p, Mg 2s and Mg 2p were detected in the broadband spectra. These elements were also found in the IC results, but the IC-detected Na^+^ and K^+^ were not found in the XPS spectra. This may be caused by the following reasons: (1) low abundances of Na^+^ and K^+^, (2) insufficient photoionization cross sections and (3) element-selective surface depletion. As for the last possibility, due to the probing depth of a few nanometers, XPS only reflects the composition of the surface, which may differ from the bulk composition determined by IC.

In addition, some carbon is present in the spectra, which is likely due to adventitious carbon contamination in the vacuum chamber as the brines have low carbonate levels as determined by IC. Note that the peak heights shown in the spectra do not represent the ratios between the elements, as the photoionization cross sections of different elements are not normalized. Nevertheless, the relative elemental ratios within the spectra can be compared to the elemental ratios of the other spectra. For example, comparing the two brines collected in different lakes (QH and MA brines), the MA brine shows a relatively strong signal for chlorine (Cl 2s and 2p) compared to O 1s (oxygen is mainly from crystallized water, sulfates and trace level organics), which means that chlorine is surface enhanced compared to oxygen, even though the bulk concentration of chloride is lower in the MA brine (Table 2). This element-selective surface enhancement of chlorine may be caused by the high level of SO_4_^2−^ in the bulk,^47^ which is higher in the MA brine (0.653 mol L^−1^) than in the QH brine (0.160 mol L^−1^). However, the SO_4_^2−^ concentration in the MA lakebed (0.124 mol L^−1^) is higher than in the QH lakebed (0.040 mol L^−1^), but the chlorine in the lakebed (Fig. 2) is smaller than in the QH lakebed. This may be caused by the different cationic compositions between the MA and QH lakebed samples. Alternatively, this could be due to the fact that the absolute SO_4_^2−^ concentration in the MA lakebed is still relatively low compared to the MA brine sample.

**Fig. 3:**
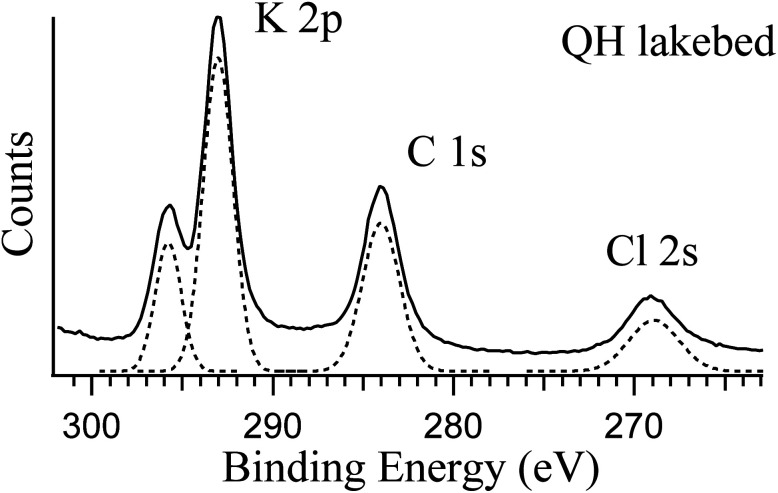
Photoemission spectra of K 2p, C 1s and Cl 2s of the QH lakebed sample at RH = 0%. The photon energy used is 584 eV. The components in dashed lines are the fittings to the spectrum.

The two lakebed samples show significantly different features. The QH sample has a strong K 2p component in addition to the other elements, which is consistent with the IC results and the fact that the QH lake is a major production source of potassium salts.^45^Fig. 3 shows the detailed K 2p spectrum of the QH lakebed sample, including the nearby peaks (C 1s and Cl 2s). A significant difference between the IC and XPS results is that the MA lakebed salt contains a high amount of Na^+^ and a relatively low level of Mg^2+^, but the XPS result only shows the Mg components. This indicates that Mg^2+^ concentrates on the surface and Na^+^ stays in deeper regions that XPS does not measure.

Concerning the crust samples, sodium (both Na 2s and Na Auger electron) was detected (Fig. 2), especially in the QH crust. Note that the Na 2s signal is weak because of the low photoionization efficiency at the photon energy used (1200 eV). By contrast, the Na Auger electron is predominant. Unlike the brine and lakebed samples, Mg^2+^ is low in the crust salts (Table 2), and thus Na^+^ is no longer depleted at the surface. Hence, the cation (Na^+^ and Mg^2+^) and the anion (Cl^−^ and SO_4_^2−^) pairs show competitive correlations. In addition, calcium (Ca 2p) is observed in the QH crust, which likely originates from the nearby desert.^48^ The carbon of the QH crust also shows interesting features, that will be discussed below.

### 3.2 Responses to water vapor

**Fig. 4:**
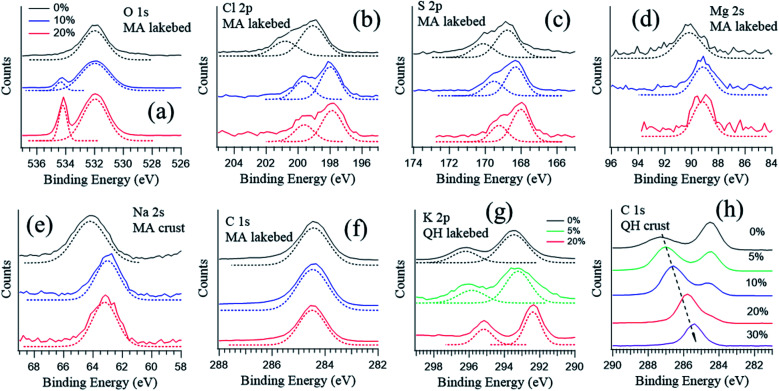
Photoemission spectra of the MA lakebed samples at RH = 0% (black), 10% (blue), and 20% (red): (a) O 1s, (b) Cl 2p, (c) S 2p, (d) Mg 2s, (e) Na 2s and (f) C 1s. Photoemission spectra of (g) K 2p of the QH lakebed samples and (h) C 1s of the QH crust at different RHs. The photon energy was varied to yield an electron kinetic energy at 300 eV.

Salt surfaces may undergo solvation or even redox reactions when water vapor is present.^49,50^Fig. 4 shows the typical photoemission spectra of the key elements at different RHs. The corresponding wide range spectra can be found in Fig. S1 in the ESI.† The most direct response to RH is the oxygen component from the gas phase H_2_O around 532 eV (Fig. 4a), which increases with increasing RH. The peaks for other displayed elements show similar shapes at different RHs (Fig. 4b–g), except for the K 2p spectrum (Fig. 4g) measured in the QH lakebed, where at 20% RH the K 2p doublets became narrower and sharper. This indicates that at 0% and 5% RH there are additional species coexisting, and at 20% RH solvation occurs and all of the K becomes free K^+^ ions.

An exception is the carbon spectra of the QH crust sample, where a carbon component shifts towards lower binding energy as the RH rises (Fig. 4h). The QH crust sample was a mixture of soil, dust and salt and dark in color, and the results imply that the mixture may undergo complicated physical and chemical processes when water is present.

### 3.3 Surface solvation at low RH

**Fig. 5:**
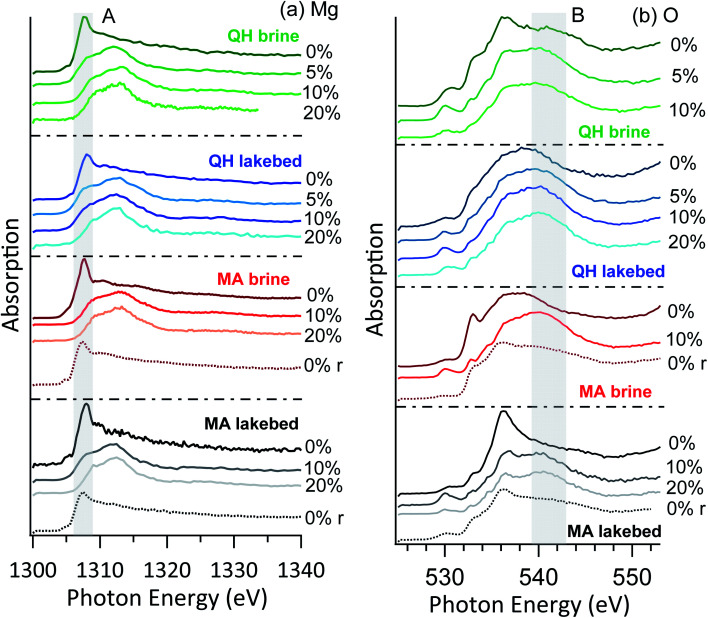
NEXAFS of (a) Mg K-edge and (b) O K-edge of the brine and lakebed samples. The *r* in the brackets indicates that the RH was reversed to 0% from high RHs.

The brine and lakebed samples show a strong abundance of Mg in the XPS spectra. Fig. 5 shows their magnesium and oxygen NEXAFS spectra at RH values ranging between 0% and 20%. Measurements were performed in the order of increasing RH, *i.e.*, from RH = 0% to 5%, 10% and 20%. In some cases (MA brine and MA lakebed), additional spectra were recorded after decreasing the RH back to 0%, and these spectra are indicated with the letter *r* representing reversed.

#### 3.3.1 Brines and lakebed salts

At RH = 0%, both the brine and lakebed samples show a strong peak (A) at the photon energy around 1307 eV (Fig. 5a). As soon as the RH rose to 5%, this feature vanished in all cases. Thus, this peak is likely attributed to the magnesium ions that are coordinated in crystalline structures. Such crystalline structures are only stable under anhydrous conditions, *i.e.*, as soon as condensed water is available on the surface these structures are destroyed. In addition, no further changes are observed in the magnesium spectrum from RH = 5% to 20%, implying that even trace levels of water vapor (RH = 5%) are enough to completely alter the physicochemical environment on the surface of the salt mixtures. Note that for these RHs, no detectable mass changes were reported from hygroscopicity measurements in a previous study,^9^ indicating that this surface solvation is only occurring in the topmost surface layers.

No major differences are seen between the brine and lakebed samples, nor between the samples from the two lakes. Thus, the RH sensitivity of magnesium is a common feature for the salts from saline lakes, including both brines and precipitated salts. This indicates that under typical environmental conditions on Earth the salt mixtures originated from saline lakes may commonly have water-doped and solvated surfaces. In the arid and cold conditions on Mars, surface salts may also interact with water vapor and consequently have solvated surfaces, as on Mars the RH commonly ranges above 5%.^51^ In the cases of the MA brine and MA lakebed samples, water dosing was closed after the measurements at RH = 20% and the RH dropped to 0%. Measurements were performed again, and the high peak feature (A) at 1307 eV above reappeared, indicating that crystallization is essentially reversible upon drying. The shape of this feature is slightly broader than the initial dry cases, which is likely due to additional hydrated magnesium components.

The oxygen NEXAFS shows distinct features in all cases (Fig. 5b), even when the magnesium spectra look similar. This could indicate that the detected oxygen atoms are in more diverse structures. Heterogeneity of the samples might also contribute to the spectral diversity. Nevertheless, as the RH increases, the spectra become more similar with a broad shoulder (region B) around 541 eV, which arises from the oxygen of condensed water.^52^

#### 3.3.2 Crust salts

**Fig. 6:**
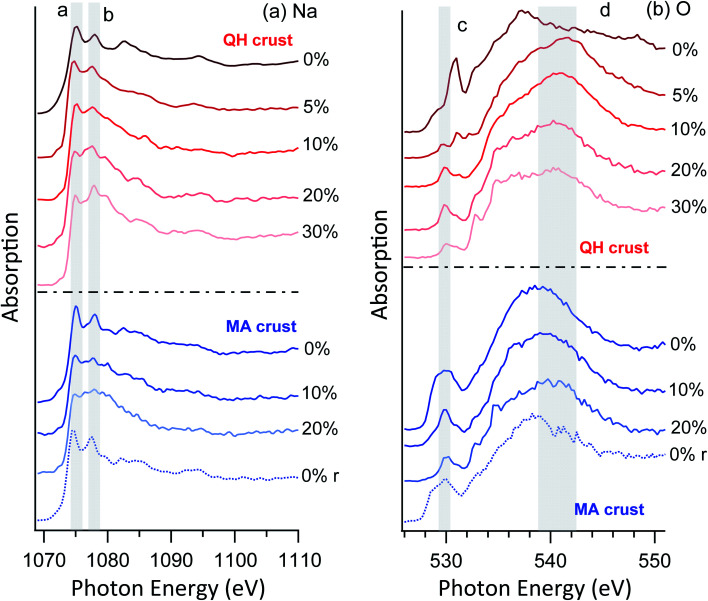
NEXAFS of (a) Na K-edge and (b) O K-edge of crust samples. The *r* in the brackets indicates that the RH was reversed to 0% from high RHs.

Unlike the brines and lakebed salts, the crust salts have a low magnesium and a high sodium concentration (Table 2). Fig. 6 shows the sodium and oxygen NEXAFS spectra of the crust salts at different RHs. Several peaks can be seen in the Na region under vacuum conditions. When the RH is raised, the peak at the lowest photon energy (a) decreases compared to the nearby peak (b), indicating surface solvation. As RH is further increased, all the peaks become less sharp as the surface is more solvated.

As for the oxygen spectra (Fig. 6b), the two crust salts show distinct features. Despite the differences between the two cases, the C–O bonds at ∼530 eV (peak c) are prominent in both cases. This is expected because the crust salts contain significant fractions of soil and dust, which may contain organic components. On increasing RH, components of condensed water appear around 541 eV (region d). When the RH is again reduced to 0%, both the sodium and oxygen spectra revert to their original appearance, confirming reversible water condensation and surface solvation. This reversibility is similar to the previously observed behavior of the surface of pure sodium acetate,^49^ but the required RH is much lower for the mixtures studied here than for a pure acetate salt. In the latter case, reversible surface solvation was only observed when the RH approaches the deliquescence RH. Furthermore, no resolvable mass changes were observed for these RHs in a previously reported hygroscopicity study of the Qaidam salts,^9^ which again supports the argument that the observed phenomena are surface specific.

## 4 Discussion

The Martian crustal compositions are similar to that of Earth, and about one third of the Martian surface was covered by oceans and lakes in the early stage of Mars development. The oceans and lakes dissolved a large quantity of minerals from the surrounding rocks, and during Martian drought periods the dissolved mineral ions precipitated and formed evaporites and salt deposits. This makes some of the saline lakes and playas on Earth considered as terrestrial analogues for Mars, such as the Qaidam Basin.

Sulfates and chlorides are among the most common salts deposited both in the Qaidam Basin and on Mars.^53^ The sulfate and chloride components sampled from the Qaidam Basin have been found to exist in a form with a high degree of hydration in spite of the arid conditions, and such hydrates have also been found on Mars.^36–38^ An important type of Martian sulfates is in the form of Mg sulfates.^54^ In a recent study on Qaidam salts, it was found that some saline lake brines contain relatively high contents of Mg^2+^ and SO_4_^2−^ but the naturally precipitated salts lack magnesium sulfates, which indicates that different mechanisms have influenced the salt compositions on Earth and Mars.^9^ Chloride salt deposits were also found on the Martian surface,^55^ but compared with Earth the coverages are smaller and mainly in or near craters.^56^ This is because the chloride salts are concentrated in ponded evaporating brines owing to their high water solubility.^57^ Martian chloride is associated with Na,^56^ similar to the NaCl dominated playa salt crusts in the Qaidam Basin.^9^ One recently discovered phenomenon on Mars is the seasonal salty water flow, termed recurring slope lineae (RSL),^42^ which is hypothesized to mainly be caused by chlorine-containing salts (particularly perchlorates) through deliquescence.^43^ However, the required water vapor levels to allow deliquescence to occur are much higher than that in the present Martian atmosphere, and thus the RSL formation mechanism and the roles of salts need to be further understood. Thus, the discoveries of highly hydrated Mg-salts and RSL raise the question of whether the occurrence of these compounds and phenomenon implies the presence of liquid water on Mars. This hypothesis is supported by the current understanding of salt–water interactions, though no liquid water has ever been found on Mars nor has it been predicted to exist in the Martian environment. In this study, we found that the Martian salt analogous are very sensitive to gas phase water, indicating that the highly hydrated Mg-salts might be formed through salt–vapor interactions. For RSL, further works need to be done to identify the possible responsible molecules and therefore study the vapor–salt interactions in more depth. The results indicate that the salt mixtures may prevailingly have partially or fully solvated surfaces under typical conditions on both Earth and Mars.

## 5 Conclusions

In this study, ambient pressure X-ray photoelectron spectroscopy (APXPS) is used to study the surface chemical environment of Qaidam salts. Regarding the ionic compositions, the XPS results generally agree with the ion chromatography results, with the exception of some elements (Na^+^ and K^+^) being absent in the surface-sensitive XPS spectra, which is likely due to their low photoionization cross sections and surface depletion. Specifically, element-selective surface enhancement of Cl^−^ is likely caused by the relatively high level of SO_4_^2−^ in the samples. Another interesting phenomenon is that Mg^2+^ is seemingly enhanced in the presence of Na^+^, which is repelled to the bulk. No major spectral changes were observed, except for the growth of the oxygen component indicating condensed H_2_O with increasing RH. The NEXAFS spectra reveal that the Mg and Na spectra change immediately as RH increases slightly, implying that the surface is solvated even when trace levels of condensed water are available. All samples show high sensitivity to RH, *i.e.*, no major differences are seen depending on the sample types and sampling locations. Thus, the highly RH-sensitive surface is a common feature for these salts, indicating that under the typical environmental conditions on Earth and Mars the salt mixtures commonly have solvated surfaces.

## Conflicts of interest

The authors declare no conflict of interest.

## Supplementary Material

EA-002-D1EA00092F-s001
