# Supplementary material for: Surface solvation of Martian salt analogues at low relative humidities
Source: Environ Sci Atmos. 2022 Jan 25;2(2):137–45. doi: 10.1039/d1ea00092f (PMC8929290; doi:10.1039/d1ea00092f)
Supplement: EA-002-D1EA00092F-s001 [file EA-002-D1EA00092F-s001.pdf]

Electronic Supplementary Material (ESI) for Environmental Science: Atmospheres.
This journal is © The Royal Society of Chemistry 2022

Supporting Information

for

Surface Solvation of Martian Salt Analogues at Low Relative Humidities

Xiangrui Kong[1*], Suyun Zhu[2], Andrey Shavorskiy[2], Jun Li[3], Wanyu Liu[3], Pablo Corral Arroyo[4], Ruth Signorell[4], Sen Wang[3] and Jan B. C. Pettersson[1*]

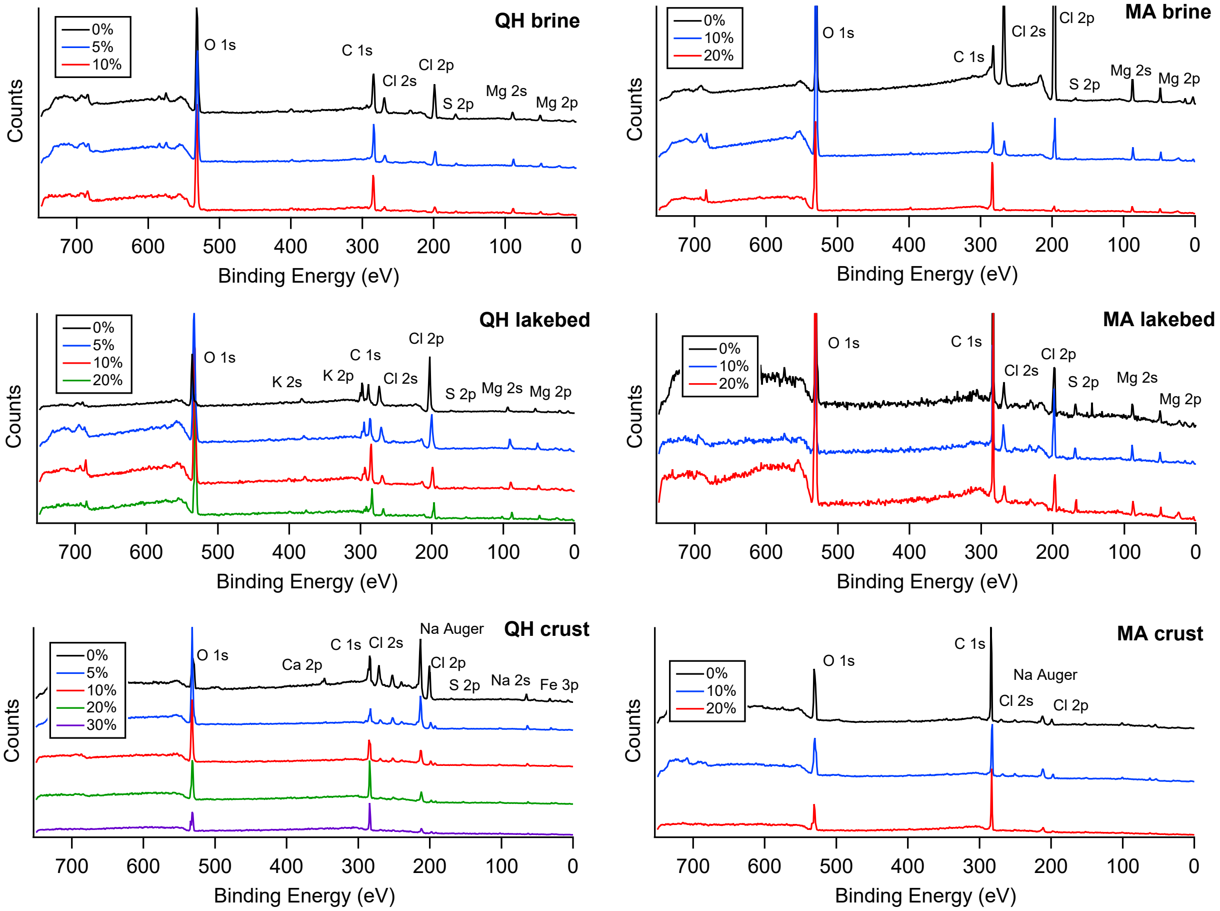

Figure S1 Broadband photoemission spectra of the six samples at RH from 0% to 30%. The used photo energy was 1200 eV.
